# Incorporation of Waste Glass as an Activator in Class-C Fly Ash/GGBS Based Alkali Activated Material

**DOI:** 10.3390/ma13173906

**Published:** 2020-09-03

**Authors:** Sasui Sasui, Gyuyong Kim, Jeongsoo Nam, Arie van Riessen, Hamin Eu, Sant Chansomsak, Syed Fakhar Alam, Churl Hee Cho

**Affiliations:** 1 Department of Architectural Engineering, Chungnam National University, Daejeon 34134, Korea; sassuikhuwaja126@gmail.com (S.S.); j.nam@cnu.ac.kr (J.N.); wp05125@naver.com (H.E.); 2 John de Laeter Centre, Curtin University, GPO Box U1987, Perth 6845, Australia; A.Vanriessen@curtin.edu.au; 3 Department of Architecture, Naresuan University, Phitsanulok 65000, Thailand; santc@nu.ac.th; 4 School of Energy Science and Technology, Chungnam National University, Daejeon 34134, Korea; fakhar_dmc@yahoo.com (S.F.A.); choch@cnu.ac.kr (C.H.C.)

**Keywords:** AAM, waste glass powder, substituted alkali solution, fly ash, GGBS, strength, hydration products

## Abstract

In this study, an alkaline activator was synthesized by dissolving waste glass powder (WGP) in NaOH-4M solution to explore its effects on the formation of alkali-activated material (AAM) generated by Class-C fly ash (FA) and ground granulated blast furnace slag (GGBS). The compressive strength, flexure strength, porosity and water absorption were measured, and X-ray diffraction (XRD) and scanning electron microscopy with energy dispersive X-ray (SEM-EDX) were used to study the crystalline phases, hydration mechanism and microstructure of the resulting composites. Results indicated that the composition of alkali solutions and the ratios of FA/GGBS were significant in enhancing the properties of the obtained AAM. As the amount of dissolved WGP increased in alkaline solution, the silicon concentration increased, causing the accelerated reactivity of FA/GGBS to develop Ca-based hydrate gel as the main reaction product in the system, thereby increasing the strength and lowering the porosity. Further increase in WGP dissolution led to strength loss and increased porosity, which were believed to be due to the excessive water demand of FA/GGBS composites to achieve optimum mixing consistency. Increasing the GGBS proportion in a composite appeared to improve the strength and lower the porosity owing to the reactivity of GGBS being higher than that of FA, which contributed to develop C-S-H-type hydration.

## 1. Introduction

To combat the high CO_2_ emissions emitted by the production of cement-based construction materials, there is an increased focus on research into “alkali-activated binders”. The production of such binders consists of alkaline activation of aluminosilicate material, such as fly ash (FA), ground granulated blast furnace slag (GGBS), metakaolin, bagasse or ashes of wood or paper [1]. However, the chemical composition of the precursor aluminosilicate material, the type and the concentration of alkali solution and the synthesis method of geopolymer binders play an important role in enhancing the properties of resultant binders [2,3]. Sodium silicate “Na_2_SiO_3_” and sodium hydroxide “NaOH” are two common activators, either combined (in a certain ratio) or used individually to manufacture geopolymer-type binders [4,5]. It is reported that, Na_2_SiO_3_, when incorporated in NaOH solution, shows promising results in improving the mechanical properties of activated precursors compared to only NaOH-activated precursors [3,6]. Generally, NaOH works as a mineralizer, which breaks the covalent bonds present in the aluminosilicate precursor material(s), therefore, releasingthe Si and Al into the system [7]. While, the Si supplied from Na_2_SiO_3_ to a solution raises the ion concentration, which increases the activation energy. The increase in activation energy accelerates the nucleation (the process in which the aluminosilicate is dissolved from precursor and forms polymeric species) and growth (a phase in which the gel begins to develop when a nuclei reaches a critical size) [8] thus forms a homogenously compact matrix with proven strength. However, the use of Na_2_SiO_3_ together with NaOH for the synthesis of geopolymer-type binders is not environmentally friendly due to its high-embodied energy. Approximately 1.51 kg CO_2_ is emitted for the production of 1 kg of sodium silicate [9,10], while the production of solid sodium silicate requires 5.371 MJ per kg, excluding the energy required for the extraction of raw material, which is not recorded [11]. This energy doubles when Na_2_SiO_3_ is used together with NaOH, as the production of NaOH pellets requires 20.50 MJ per kg [12]. Based on these figures, it is clear that alternative alkaline solutions need to be identified to enable manufacture of alkali-activated material (AAM) with lower environmental impact.

One approach that is more sustainable is to use amorphous soda-lime glass as a source of Si. This glass, with a chemical composition of SiO_2_ (65–75 wt.%) and Na_2_O (12–15 wt.%), is available as a waste and a potential source of Si [13]. Glass rich in alkalis can be used in conjunction with NaOH instead of sodium silicate to produce an alkali activator with moderate environmental impact due to greenhouse gas emission and energy consumption [14]. Additionally, recycling the waste glass reduces the need for landfill as this material is non-biodegradable, which constitutes an issue for many municipalities [15].

Generally, glass can be dissolved in a caustic environment, with the pH of the aqueous alkali defining the mechanism and degree of glass corrosion. The degree of formation of soluble silicates also increases with the higher temperature [16,17]. Glass being a source of soluble silicates has attracted attention as an activator in manufacturing alkaline-activated binders. Specifically, use of soda-lime glass as a component of alkali activator has been explored in several studies. Torres-Carrasco and Puertas [18] in their study concluded that the waste glass powder (WGP) + NaOH-10M (15 g/100 mL) activator can be used to replace NaOH-10M + 15% Na_2_SiO_3_ solution, as their study revealed similar effects in enhancing the strength and microstructure of Class-F FA geopolymer cement. The study conducted by Carrasco et al. [19] showed that Class-F FA, when activated by WGP + NaOH-10M (15 g/100 mL), showed greater strength than NaOH-10M + 15% Na_2_SiO_3_ and NaOH (8M)-activated Class-F FA. However, the reactivity of WGP+NaOH-10M was observed to be similar to that of NaOH-10M + 15% Na_2_SiO_3_ and thus, they considered the waste glass-based alkali as an alternative to sodium silicate solution. When waste glass dissolved in NaOH + Na_2_CO_3_ was used to activate slag, the strength increased with increasing glass content in NaOH + Na_2_CO_3_, i.e., up to 25 g/100 mL, and formed reaction products similar to that of NaOH + Na_2_CO_3_ + Na_2_SiO_3_ [20]. Tchakouté et al. [21] synthesized a waste glass-based alkali activator by dissolving glass powder and NaOH pellets (molar ratio: SiO_2_/Na_2_O = 1.5 and H_2_O/Na_2_O = 10) in 200 mL water and declared this solution to be suitable as an alkaline activator for a metakaolin-based geopolymer. Given that the research on a waste glass-based alkali activator is limited and no research has been conducted on the influence of this activator on the properties of high calcium (Ca) alkali-activated material (AAM), it suggests that the research reported here is warranted. Class-C FA and GGBS composed of high Ca was selected for our previous study [6]. It was determined that the Class-C FA compared to GGBS is less reactive when activated by NaOH-4M solution, but when these materials were activated by NaOH+Na_2_SiO_3_ solution, the dissolution of FA and GGBS was enhanced. Therefore, in this study, the Class-C FA and GGBS were again selected as precursor materials to explore the influence of a waste glass-based alkali activator on their dissolution. Furthermore, the optimum quantity of glass powder needed to synthesize the alkaline solution for high calcium AAM, i.e., Class-C FA/GGBS, is not known. Therefore, this study aims to explore the feasibility of using WGP-alkaline solution for Class-C FA/GGBS-based AAM.

## 2. Materials and Methods

### 2.1. Materials, Mixture Proportions and Samples Synthesis Method

**Figure 1:**
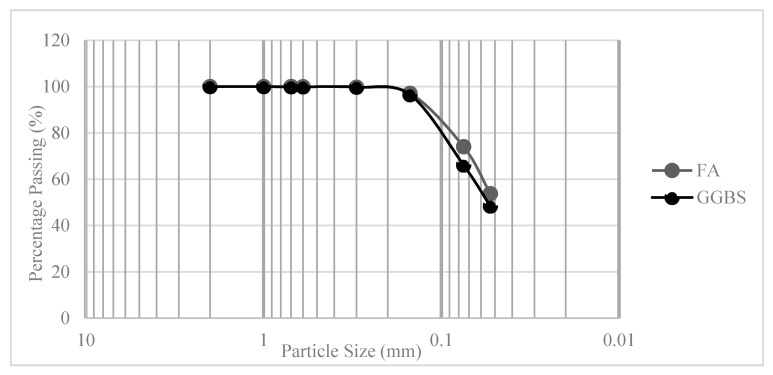
Particle size distribution of fly ash (FA) and ground granulated blast furnace slag (GGBS).

In this study, Class-C fly ash (FA), as per ASTM C618-19 [22], and ground granulated blast furnace slag (GGBS) were used as precursor materials (see Figure 1 for particle size). For synthesizing the waste glass-based alkaline solution (WGA), soda-lime waste glass powder (WGP) obtained in a crushed form from “Indong G.R.C” and commercial NaOH pellets (assay 97%) were used. The soda-lime glass was supplied in three different colors, brown glass (BG), green glass (GG) and transparent glass (TG). The chemical composition of precursor materials, i.e., FA and GGBS, and waste glass powder (WGP), i.e., BG, GG, TG (see Table 1), was obtained by energy dispersive X-ray analysis using a Tescan focused ion beam scanning electron microscope (FIB-SEM) LYRA3 XMU (TESCAN, Brno Czech Republic, EU). Samples were coated with a thin layer of Pt prior to analysis and the average of three point-mode measurements was taken as the composition.

As noted from the chemical composition, the BG and TG contain Si above 30 wt.% and relatively high Ca content, whereas the GG comparatively has lower Al and Ca content but is still rich enough in Si to make it eligible to be used in this study. These three different colored glass particles were mixed in equal proportions to make up 2 kg, then ground in a ball mill for 30 min. The powder obtained after milling was passed through an ASTM designated sieve #200 (i.e., particle size below 75 µm) and used to prepare WGA. To synthesize the WGA, NaOH (4M) solution was prepared by dissolving NaOH pellets in distilled water and cooled to ambient temperature for 24 h prior to use. It has been reported that dissolution of glass in high molarity NaOH (~10M) may accelerate the dissolution rate of aluminosilicate precursors, but can lead to disintegration of the geopolymer network after 28 days of curing [5]. Thus, NaOH with molarity 4 was selected for this study considering the hardening of AAMs which are high in calcium (Ca) [23,24,25]. The sieved WGP was dissolved in the NaOH (4M) solution in three different proportions, i.e., 10, 20 and 30 g per 100 mL of NaOH (4M) solution. Following is a description of the dissolution method used in this study.

The aforementioned specified quantities of glass powder were added to 100 mL NaOH (4M) solutions. The mixture was stirred using a hot plate magnetic stirrer for four hours at 300 rpm with the temperature of the plate set to 60 °C. After four hours of mixing, the solution was cooled to ambient temperature for 48 h to allow continued dissolution of silica from the glass powder. The solution was then filtered and left to equilibrate for 24 h before being used for experiments.

The FA and GGBS were combined in various ratios and activated with synthesized WGA alkaline solutions (Table 2). Dry precursor materials were first mixed in a Harbor mixer at low speed for 10 min, then alkaline solution was added gradually to the dry mix (at liquid/solid ratio = 0.4) and the blend was mixed at moderate speed for five minutes. To achieve the same consistency of pastes, i.e., approximately the same slump (slump test performed as recommended by Tan and Bernal [26] in their study), water was added slowly to the blends as required and mixed for a further 15 min. The water added to each paste varied, as discussed in Section 3.2. The prepared paste was then poured in metallic molds, compacted for 2.5 min using a vibrating compactor and then sealed in plastic bags to avoid the loss of moisture from the paste. These molds were initially kept in the ambient laboratory environment for two hours and then oven-cured at 60 °C for 24 h. Although high Ca AAM materials can be cured at ambient temperature, in this study, curing at 60 °C for 24 h was chosen to enable maximum dissolution and geopolymerization prior to demolding and aging [27]. After 24 h of oven curing, the samples were unsealed and cooled to ambient temperature for 2 h before demolding. Samples were then aged for 28 days at 22 (±5) °C temperature and 50 (±5) % relative humidity.

### 2.2. Experimental Plan

The chemical composition of the alkaline solution synthesized from waste glass, i.e., WGA-10, WGA-20 and WGA-30, was determined using Inductively Coupled Plasma—Atomic Emission Spectroscopy (ICP-AES) (model Optima 7300 DV, PerkinElmer, Inc., Waltham, MA, USA). To determine mechanical properties of the AAM, compression testing in accordance with ASTM C109/C109M-16a [28] and flexural testing in accordance with ASTM C348-18 [29] were conducted (three of each sample batch were tested to obtain an average strength value). For determination of physical properties, i.e., apparent porosity and water absorption, use of Archimedes’ principal as described in ASTM C20-00 [30] was applied to the specimens. For the raw FA and GGBS and for the samples (collected after compression test then ground to a fine powder), XRD patterns were obtained using an X-ray Diffractometer (Bruker AXS D8 ADVANCE, Bruker AXS GmbH, Karlsruhe, Germany), over the 2θ range 5–100° with a wavelength of Cu target (λ = 0.15406 nm). To explore the microstructure, secondary electron images were obtained using 5.0 keV primary electrons, and to determine the distribution of major elements in the samples, EDS was carried out at 10.0 keV using a FIB-SEM LYRA3 XMU (TESCAN, Brno, Czech Republic) on Pt-coated samples collected from the core of 50 mm^3^ samples used for the compression test.

## 3. Experimental Results and Discussion

### 3.1. Chemical Composition of WGA-10, WGA-20 and WGA-30 Solution

**Figure 2:**
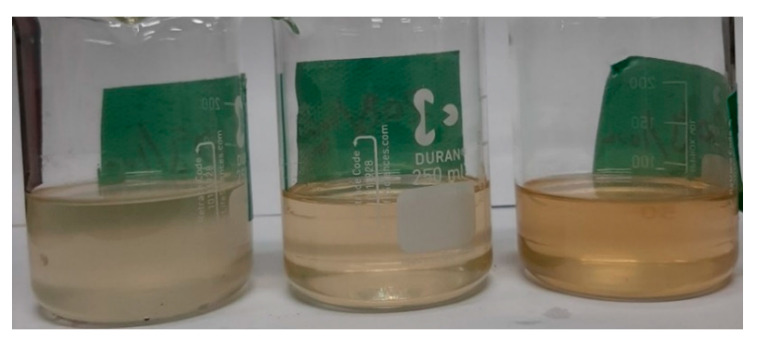
Glass powder-based alkaline solution: WGA-10, WGA-20 and WGA-30.

ICP-AES results for major elements, i.e., Si, Na, Al, Ca and Mg, in solutions obtained after dissolving glass powder (WGA-10, WGA-20 and WGA-30) are presented in Table 3. From the results, it can be seen that the leaching of elements from glass was increased with increasing glass content from 10 to 30 g in 100 mL NaOH-4M solution. Additionally, non-congruent dissolution of Mg was observed in WGA-30 solution, indicating the fact that more dissolution occurred, releasing most of the Mg from the glasses. As the glass content in the NaOH-4M solution increased, the color changed, as shown in Figure 2. The solution was observed to be darker and more viscous with increasing glass content, consistent with an increase of Si concentration in NaOH solution.

### 3.2. Addition of Water to FA/GGBS Blends

**Figure 3:**
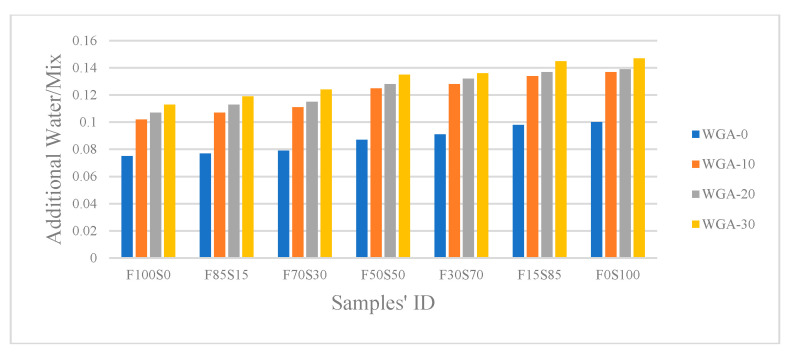
Additional water for paste mixes.

As mentioned previously, each FA/GGBS paste from each batch was prepared to obtain the same mixing consistency, so water was added to the blend of FA/GGBS and alkaline solution, according to the demand of each paste. The ratio of water to the mix composed of FA/GGBS and alkali solution (Additional water/Mix) plotted in Figure 3, shows that each FA/GGBS composite demanded more water with the increasing WGP content in NaOH solution. This can be attributed to the viscosity of the solution, which was observed to increase as the glass content added to NaOH was increased. In general, the water demand for composites increases when the chemical reactivity of precursors increase [31]. In this study, the FA/GGBS was initially mixed with prepared alkaline solution for 5 min, it was observed that there was an increase in water demand with increasing glass content in NaOH solution, i.e., WGA-10, WGA-20, WGA-30 (See Figure 3). This supports the proposition that with more dissolved Si in the NaOH, the higher the reactivity of precursors, which in turn increased the water demand. Furthermore, it can be seen in Figure 3 that the water demand of the FA/GGBS composite in each batch increased with increasing GGBS, indicating the high reactivity of GGBS used in this study.

### 3.3. Mechanical Performance: Compressive Strength and Flexural Strength

**Figure 4:**
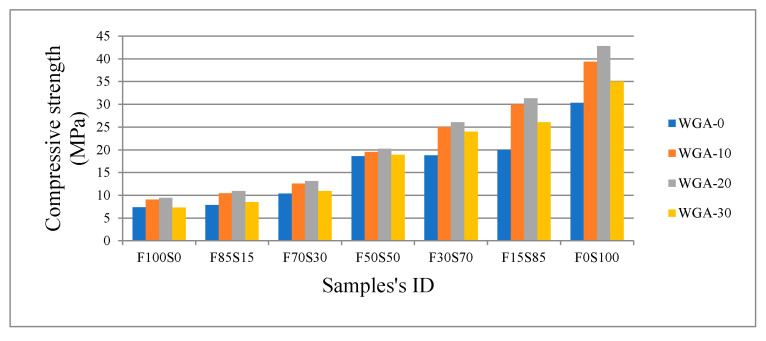
Compressive strength versus FA/GGBS and WGA solution.

**Figure 5:**
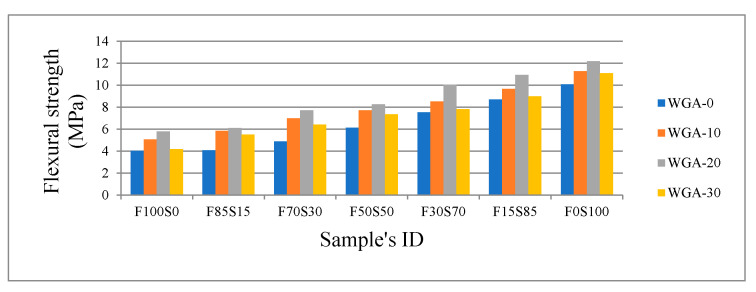
Flexural strength versus FA/GGBS and WGA solution.

The compressive strength and flexural strength developed by FA/GGBS samples in each batch is shown in Figure 4 and Figure 5, respectively. For all FA/GGBS combinations, compressive strength and flexural strength increased as WGP increased from 0 to 20 g/100 mL NaOH, i.e., from WGA-0 to WGA-20. The strength was observed to decrease when going from 20 to 30 g/100 mL NaOH, i.e., from WGA-20 to WGA-30. The increase of strength with increasing WGP from 0 to 30 g/100 mL in NaOH solution is believed to be due to the increase of Si in the solution, which promotes the leaching of elements from precursor materials, i.e., FA and GGBS, leading to more gel formation and improved strength. The decrease in strength on activation with WGA-30 solution is believed to be due to the formation of porous gel resulting from the change in the mechanism of geopolymerization when the precursors are activated by alkaline solution with high concentration of Si [32]. As stated by Duxson and Fernández-Jiménez [33] in their study, the variation of the Si/Na ratio modifies the degree of polymerization of species dissolved in an alkaline solution, which significantly impacts the properties of gel formed after activating the precursors in these solutions. During the polycondensation process in a paste activated by a solution with increased Si concentration, i.e., WGA-30, the water is able to accumulate in various region of gel, creating micropores that remain after hardening or setting of the gel, resulting in a weaker structure [32,34,35]. Moreover, the increasing viscosity of the solution with increasing WGP content in NaOH solution (WGA-30) is also thought to contribute to strengthening the reduction of FA/GGBS samples as it increases the water demand of the system (see Figure 3). The increase of additional Water/Mix ratio in WGA-10 and WGA-20-activated pastes eases the polymerization as ion mobility enhances, resulting in increased mechanical strength [36]. However, the excessive water in a system promotes the dissolution of precursor materials, but with increasing ion concentration in a medium, decelerates the geopolymerization, which limits the ion mobility and therefore limits the coagulated gel to form in a matrix [37].

It is noticeable that the GGBS is far more reactive than the FA for all alkali solutions trialed. The greater reactivity and associated composition of the GGBS resulted in improved strength compared with samples made from only FA or a high proportion of FA. For instance, when using the NaOH only (WGA-0), the strength increased by 313% when comparing 100% FA to the 100% GGBS sample. When the WGA-20 was used to activate the 100% FA sample and 100% slag sample, there was a 356% increase in strength. Even though samples activated with WGA-30 showed a slight strength decrease compared to WGA-20-activated samples, there was still a 381% increase in strength for WGA-30-activated samples when comparing 100% FA with 100% GGBS samples. These impressive strength increases clearly indicate the benefits of using the WGA activators with GGBS precursors.

### 3.4. Apparent Porosity and Water Absorption

**Figure 6:**
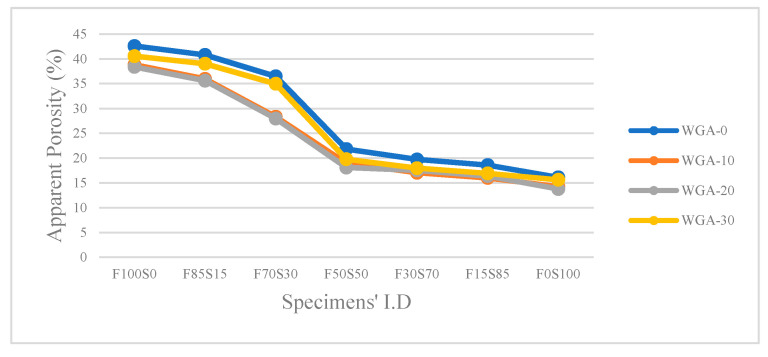
Apparent porosity of WGA-activated FA/GGBS samples.

**Figure 7:**
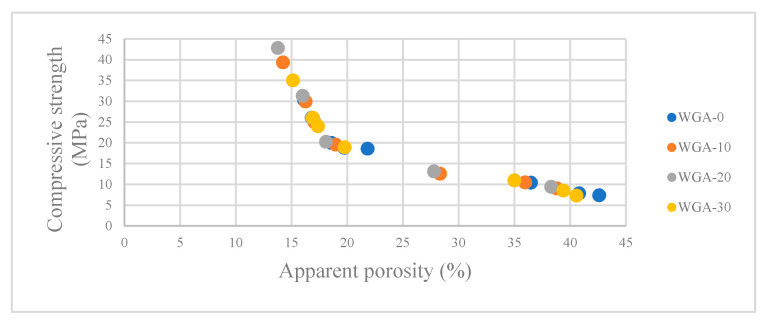
Apparent porosity versus compressive strength.

The apparent porosity and apparent porosity versus compressive strength results of WGA-activated FA/GGBS composites are presented in Figure 6 and Figure 7, respectively. From the results plotted in Figure 6, it can be seen that the highest porosity was observed for FA/GGBS samples activated by WGA-0, and it reduced when activated by WGA-10, WGA-20 and WGA-30. Furthermore, the results revealed that increasing the GGBS content in FA/GGBS composites also led to the reduced porosity. As expected, these results show that the porosity is inversely proportional to the compressive strength (Figure 7). The porosity in a sample is created when water is expelled during the geopolymerization process and is trapped in the microstructure. However, from the additional Water/Mix ratio graph (Figure 3), it was noted that the water added in WGA-10, WGA-20 and WGA-30 batches was comparatively more than the WGA-0 batch, but the porosity of WGA-0 samples is higher than WGA-10, WGA-20 and WGA-30 samples. This behavior is associated with the amount of hydroxyl ions supplied from the additional H_2_O to the system and alkali metal ions, i.e., Na supplied with increasing WGP content in NaOH solution, which accelerates the alkali activation. The impact of this is to form either more gel or a gel with optimized Si/Al, creating a matrix with strong inter-particle bonding and reduced porosity [38,39]. Thus, the high porosity in WGA-0 samples suggests that incomplete geopolymerization and resulting porosity is associated with the voids between unreacted particles of FA and GGBS. Whereas, the increased porosity of WGA-30-activated samples compared with WGA-20- and WGA-10-activated samples indicated that the matrix was negatively impacted due to the high water-demand forming porous gel, as explained in Section 3.3.

Consequently, the FA/GGBS ratio also significantly influenced the porosity (Figure 6). When the composite consists of high FA content, i.e., from 100% to 70% (F100S0–F70S30), the porosity is high in each batch. A further decrease in the proportion of FA below 70% (increase in proportion of GGBS above 30%) led to a sudden decline in porosity. There was a 62.17% decrease in porosity from the highest porosity sample (F100S0 activated by WGA-0) to the low-porosity sample (F0S100 activated by WGA-0). A similar trend was observed for samples activated by WGA-10, WGA-20 and WGA30 solutions. This dramatic decrease in porosity again highlights the benefit of using GGBS over FA. The increasing amount of Ca from GGBS in the samples leads to the production of either a C-S-H-type gel or a C-A-S-H-type gel in a matrix, which promotes more cross-linking in the binder than the geopolymer gel, i.e., N-A-S-H gel [40], resulting in reduced porosity.

**Figure 8:**
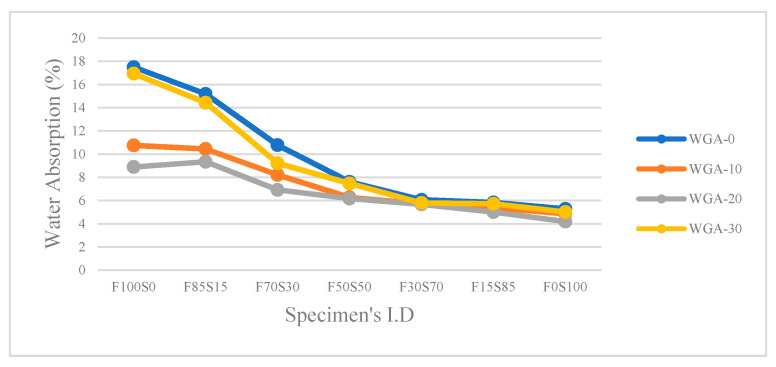
Water absorption of WGA-activated FA/GGBS samples.

Results of samples’ water absorption measurements are presented in Figure 8. Water absorption data indicate the presence of void space in a sample due to the open porosity. Results confirm the presence of higher open porosity in FA/GGBS composites activated by WGA-0 solution. Results also confirm the decline in water absorption with increasing GGBS in FA/GGBS composites, confirming the aforementioned fact that increasing Ca from GGBS reduced porosity due to the formation of C-S-H or C-A-S-H gels in the matrix. Figure 8 also reinforces previous observations that WGA-30-activated samples have greater open porosity and therefore greater water absorption due to these samples demanding more water during mixing.

### 3.5. XRD Diffraction

**Figure 9:**
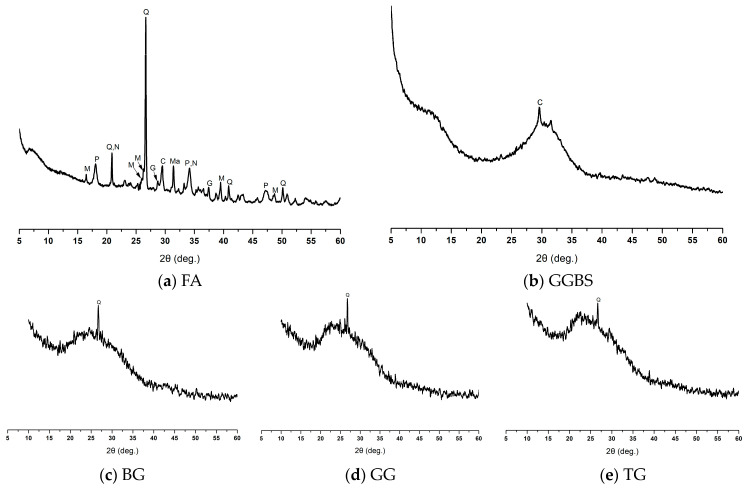
XRD patterns of precursors; (**a**) FA, (**b**) GGBS and three colored WGP; (**c**) Brown glass (BG); (**d**) Green glass (GG); (**e**) Transparent glass (TG). Note: Q = quartz (00-046-1045 > SiO_2_), M = mullite (00-015-0776 > Al_6_Si_2_O_13_), Ma = magnetite (01-079-0418 > Fe_3_O_4_), C = calcite (01-071-3699 > CaCO_3_), P = Portlandite (00-004-0733 > Ca(OH)_2_), N = sodium magnesium aluminum silicate (00-047-1498 > Na_1.74_MgO_0.79_Al_0.15_Si_1.0604_), G = gehlenite (00-035-0755 > Ca_2_Al_2_SiO_7_).

XRD patterns of precursors, i.e., FA and GGBS, and three different colored WGP are plotted in Figure 9a–e. FA was found to consist of the crystalline phases of quartz, mullite, magnetite, calcite, Portlandite, sodium magnesium aluminum silicate and gehlenite (Figure 9a). The presence of calcite, portlandite and gehlenite suggests that the Ca present in FA is mostly crystalline in nature. GGBS on the other hand was found to be composed of mainly amorphous phases, as the pattern (Figure 9b) shows only a small peak of calcite along with the broad hump between 2θ = 23–37.5°. The XRD patterns of WGP, i.e., BG (Figure 9c), GG (Figure 9d) and TG (Figure 9e) consist of amorphous phases, as shown by a broad hump between 2θ = 17.5–32°, and crystalline quartz, as shown by a peak at 2θ = 26–27°.

**Figure 10:**
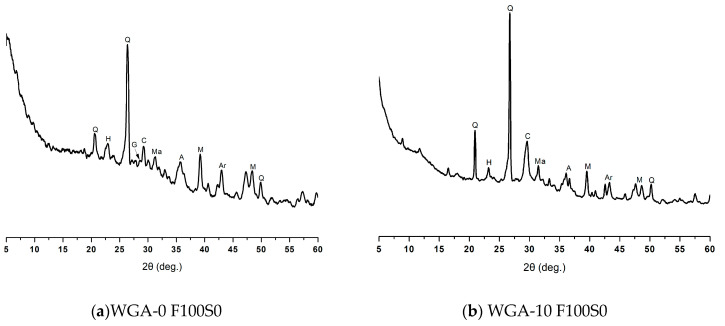

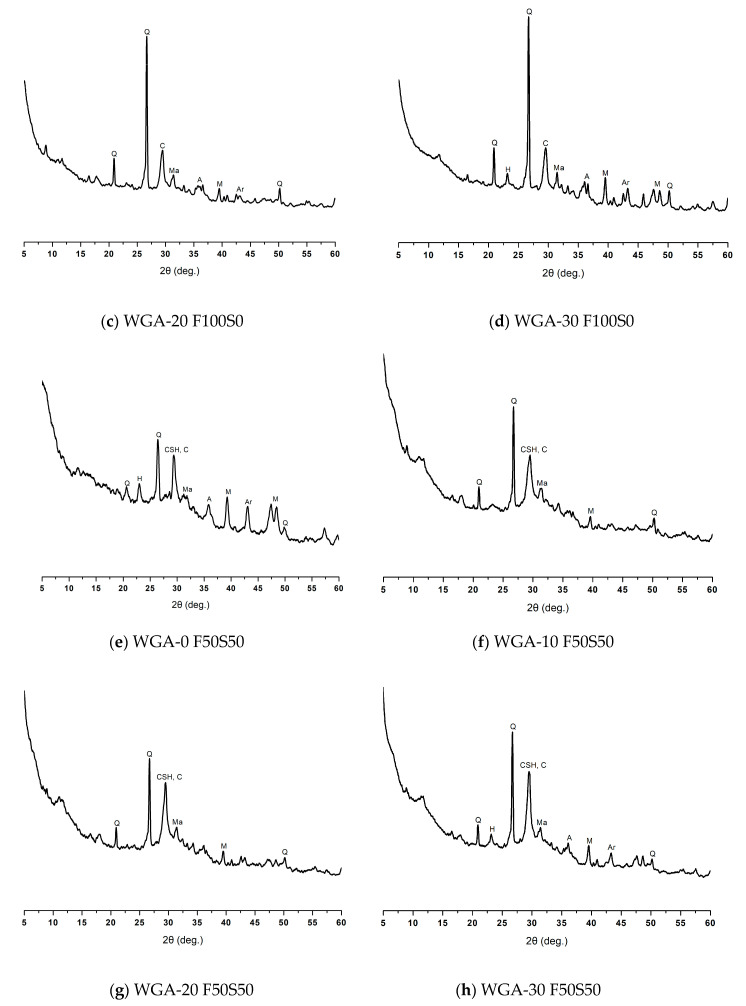

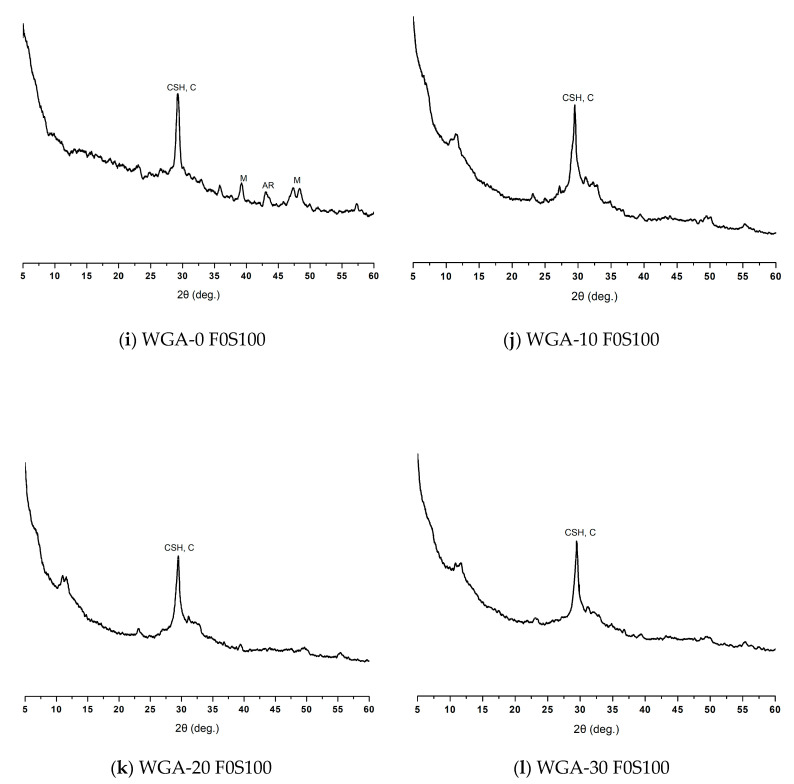
XRD diffraction pattern of; (**a**) WGA-0 F100S0, (**b**) WGA-10 F100S0, (**c**) WGA-20 F100S0, (**d**) WGA-30 F100S0, (**e**) WGA-0 F50S50, (**f**) WGA-10 F50S50, (**g**) WGA-20 F50S50, (**h**) WGA-30 F50S50, (**i**) WGA-0F0S100, (**j**) WGA-10 F0S100, (**k**) WGA-20 F0S100, and (**l**) WGA-30 F0S100. Note: Q = quartz (00-046-1045 > SiO_2_), Ma = magnetite (01-079-0418 > Fe_3_O_4_), M = mullite (00-015-0776 > Al_6_Si_2_O_13_), CSH = calcium silicate hydrate (00-22-0600 > 2CaSiO_3_.3H_2_O), C = calcite (01-071-3699 > CaCO_3_), Ar = aragonite (01-076-0606> CaCO_3_), H = hydrosodalite (00-011-0401 > Na_4_Al_3_Si_3_O_12_(OH)), A = albite (00-009-0466 > NaAlSi_3_O_8_), G = gehlenite (00-035-0755 > Ca_2_Al_2_SiO_7_).

The XRD patterns of FA/GGBS samples F100S0, F50S50 and F0S100 activated by WGA-0, WGA-10, WGA-20 and WGA-30 solutions are plotted in Figure 10. Sample F100S0 activated by WGA-0 solution showed the crystalline phases of quartz, magnetite, mullite, calcite and gehlenite (Figure 10a), which are similar to those identified in raw FA. Aragonite, albite and hydrosodalite were also present as new phases, which were observed to be reduced when the sample F100S0 was activated by WGA-10, WGA-20 and WGA-30 solutions (Figure 10b–d), indicating that the crystals partially changed to amorphous phase. The sample F100S0 activated by WGA-10, WGA-20 and WGA-30 solutions (Figure 10b–d) showed comparatively strong peaks of quartz between 2θ = 26–27° and 2θ = 20.5–21.1°, as well as the calcite peak between 2θ = 28.5–30°. Generally, the presence of quartz does not interrupt the geopolymerization process and remains present as fine particles in the microstructure. These fine particles may act as a reinforcement, creating a barrier against crack propagation, resulting in higher mechanical properties [41]. The calcite peak for the F100S0 samples activated by WGA-10, WGA-20 and WGA-30 solutions suggest the formation of a more calcium-based reaction product [42].

As GGBS goes from 0 to 50 wt.% (sample F50S50, as presented in Figure 10e–h), the crystalline peaks are reduced and the broad hump increases (2θ = 25–38°), suggesting an increase in the formation of amorphous phases. Additionally, with the increase of GGBS, the F50S50 samples showed the presence of C-S-H along with the calcite peak due to the presence of reactive Ca supplied from GGBS. Furthermore, the peaks of magnetite, mullite and aragonite reduced when F50S50 samples were activated by WGA-10, WGA-20 and WGA-30 solutions (Figure 10f–h). No traces of albite or hydrosodalite are observed for WGA-10- and WGA-20-activated F50S50 samples (Figure 10f,g), and the peaks of these phases reduced in the WGA-30-activated F50S50 sample (Figure 10h).

The XRD patterns of samples F0S100 (Figure 10i–l) show less crystalline reflections compared to the patterns from F100S0 and F50S50 samples, exhibiting similar features to the XRD pattern of GGBS. The sample WGA-0 F0S100 in Figure 10i shows the presence of mullite, aragonite and calcite with the traces of C-S-H, and when activated by WGA-10, WGA-20 and WGA-30 solution, the peaks of mullite and aragonite are not present, suggesting a reduction in crystalline phases. While, the hump at 2θ = 25–38° exhibited for the samples activated by WGA-10, WGA-20 and WGA-30 F0S100 indicates the formation of amorphous gel phases upon the activation by WGP-dissolved alkaline solution.

### 3.6. SEM/EDX Micrographs

**Figure 11:**
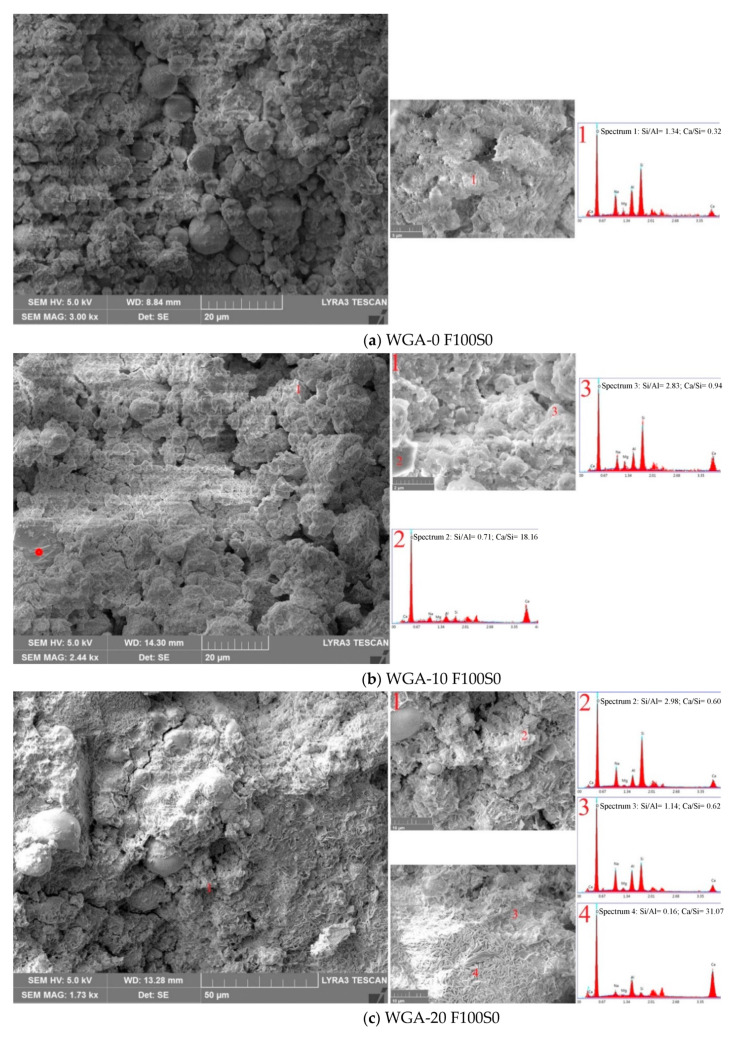

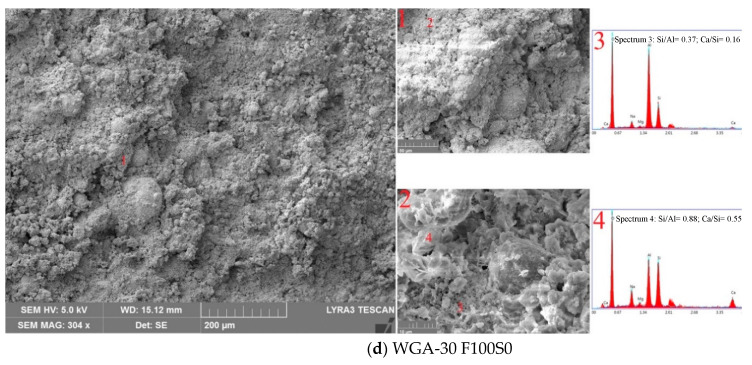
SEM Micrographs and EDX spectra of sample F100S0 activated in (**a**) WGA-0, (**b**) WGA-10, (**c**) WGA-20 and (**d**) WGA-30 solution.

**Figure 12:**
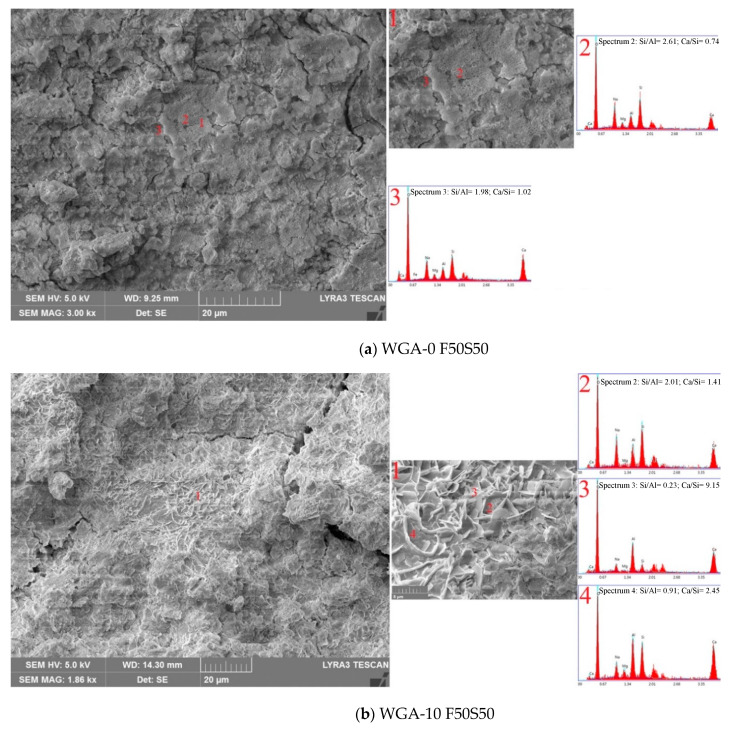

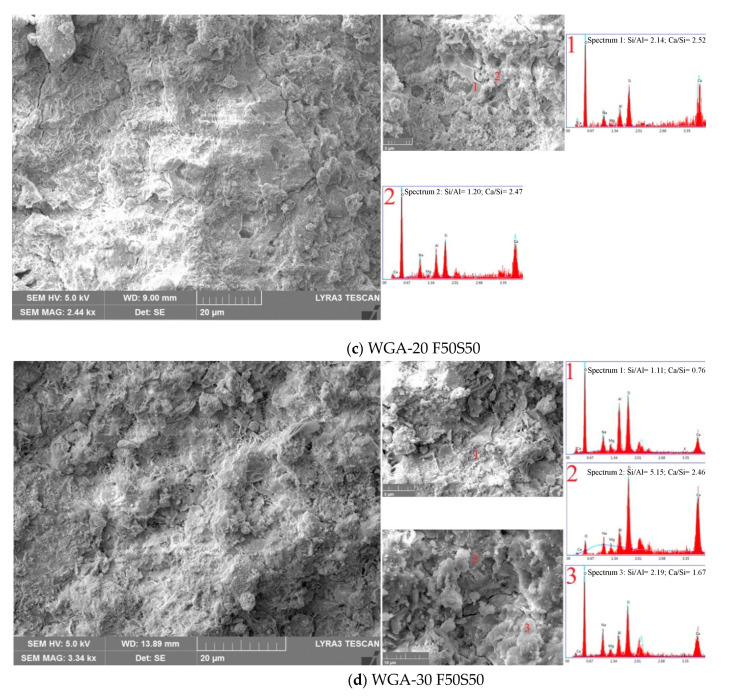
SEM Micrographs and EDX spectra of sample F50S50 activated in (**a**) WGA-0, (**b**) WGA-10, (**c**) WGA-20 and (**d**) WGA-30 solution.

**Figure 13:**
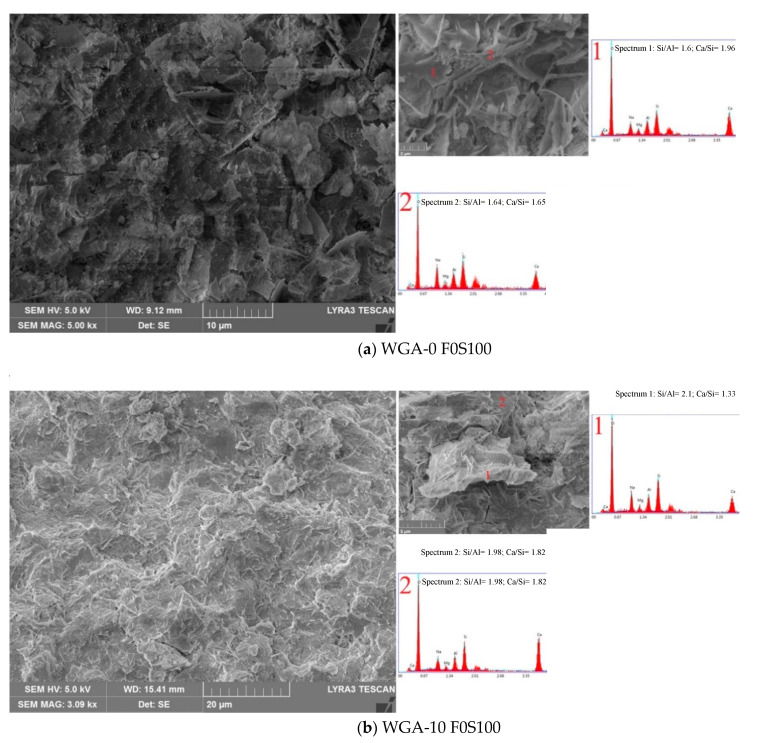

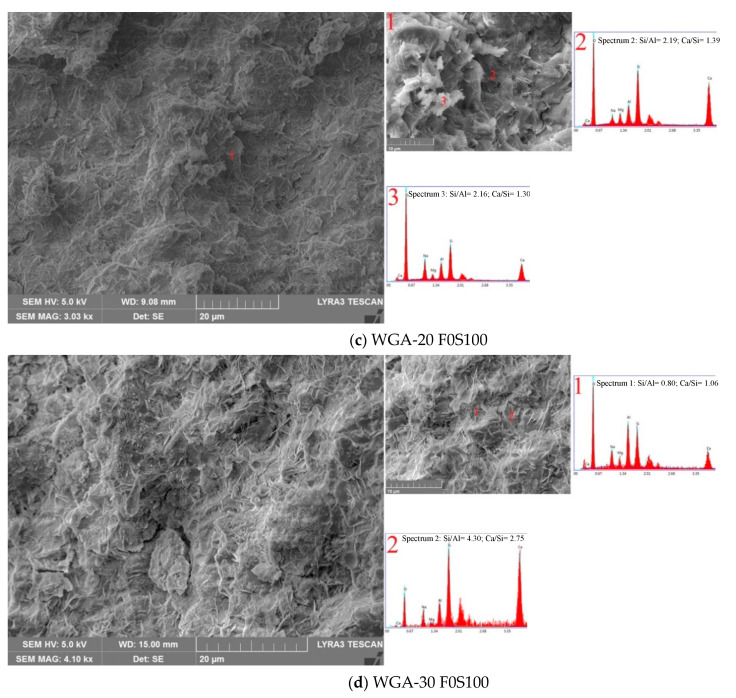
SEM Micrographs and EDX spectra of sample F0S100 activated in (**a**) WGA-0, (**b**) WGA-10, (**c**) WGA-20 and (**d**) WGA-30 solution.

The SEM micrographs and EDX spectra of samples F100S0, F50S50 and F0S100 from each batch are shown in Figure 11, Figure 12 and Figure 13. Sample F100S0, activated by WGA-0, WGA-10, WGA-20 and WGA-30 (Figure 11), shows unreacted FA particles encapsulated in binder, suggesting only partial dissolution of FA in the matrix. Comparatively, the sample WGA-0 F100S0, shown in Figure 11a, appeared less dense with maximum undissolved FA particles surrounded by the gel, and exhibits the lowest strength. The formation of gel in this sample is heterogeneous, as seen in the magnified view of the matrix, while the EDX spectrum of this gel is composed of Si, Al and Na, with negligible Ca, forming the N-A-S-H-type reaction product [43]. When the FA (F100S0) was activated by WGA-10 solution (see Figure 11b), the amount of gel phases increased, suggesting increased dissolution of FA. A crystal (marked with a red dot on 20 µm scaled micrograph and as “2” on 2 µm scaled micrograph in Figure 11b) composed mainly of Ca (see spectrum 2 in Figure 11b) is believed to be calcium hydroxide, i.e., Ca(OH)_2_ (CH), based on the morphology that was found in the microstructure [44,45]. From the micrographs, it is apparent that there are less CH crystals present than gel “3” which is composed of mainly of Si, with sufficient Na, Al and Ca. In this gel, the Si/Al ratio increased relative to the WGA-0 F100S0 sample and the presence of low Ca, suggesting the formation of Ca-substituted N-A-S-H, i.e., (N,C)-A-S-H [46,47], which is denser than N-A-S-H observed in WGA-0 F100S0, thus improving the strength of the matrix. Whereas, the contribution of Ca in forming gel is an indication of increased dissolution of FA when activated by WGA-10. In Figure 11c, the matrix of sample WGA-20 F100S0 appears to have a greater amount and denser gel than that observed in WGA-0 F100S0 and WGA-10 F100S0 samples, filling the spaces between undissolved FA particles. Three different gels were observed in this sample: the gel marked as “2” is rich in Si with medium-intensity X-ray peaks of Na and Al, and negligible Ca forming Si-rich N-A-S-H gel, thus making the gel dense. The second gel marked as “3” comparatively covered less area in the matrix and is the same as N-A-S-H gel but with comparatively reduced Si and increased Al, while the gel marked as “4” covered most of the area of the matrix and the spectrum of this gel is mainly composed of high Ca and medium Al, with minor traces of Na and Si. It can be assumed that this gel is a modification of the CH crystal formed in the WGA-10 F100S0 sample, which reacts with Al to form calcium aluminate hydrate, i.e., C-A-H-type gel, with improved density and also the strength [48,49]. The sample WGA-30 F100S0 in Figure 11d shows the matrix consisting of maximum gel formation along with some undissolved FA particles. Although this matrix formed the maximum amount of gel, it was observed to be disintegrating, and thus weakening, the matrix, therefore lowering the strength. The lower strength of this matrix is associated with the formation of porous matrix, as observed in point “2” of Figure 11d. As shown in the spectrum of porous gel “3”, which is mainly composed of Al and Si showing a high Al peak, lowering the Si/Al ratio, indicating the possible formation of alumina-rich zeolites responsible for reduced strength [7]. In the same matrix, composition of the gel marked as “4” is rich in both Al and Si, with low Na and negligible Ca, indicating the formation of N-A-S-H gel but with a lower Si/Al ratio. Ruiz-Santaquiteria et al. [35] in their study explain the reason why their matrix formed Al-rich N-A-S-H gel and Al-rich zeolites when water was increased in a system due to the hydrophilic nature of Al and hydrophobic nature of Si. Furthermore, the development of porous gel is associated with the accumulation of excessive water in the matrix, which on hardening leaves micropores throughout the matrix. From the aforementioned discussion, it is noted that the Ca in all F100S0 samples does not contribute to forming the C-S-H- or C-A-S-H-type gels and the reason could either be the low Ca environment or unreactive Ca present in the medium. This is supported by the XRD results, which showed no trace of C-S-H in all of the F100S0 matrices (See Figure 10). Furthermore, the micrographs and EDX results clearly show that the dissolution of FA increased as the WGP content in NaOH solution (10–30 g/100 mL NaOH) increased, resulting in greater gel formation.

The micrographs of sample F50S50 shown in Figure 12 are denser than the F100S0 samples (Figure 11), resulting in improved strength. As observed in Figure 12a, the sample WGA-0 F50S50 exhibits a comparatively loose matrix composed of two different gel compositions with few unreacted GGBS and FA particles. The gel marked as “2” in light gray covered a large proportion of the matrix composed of medium Ca and Al and high Si and Na, suggesting the presence of Ca-substituted N-A-S-H gel or (N,C)-A-S-H. Whereas, the gel marked as “3” showed higher levels of Ca with reduced Si and Na, while Al remained the same as in spectrum “2”, leading to the formation of C-A-S-H- or C-N-A-S-H-type gels [46,47]. The sample WGA-10 F50S50 in Figure 12b exhibits a denser gel than that observed for the WGA-0 F50S50 sample. Three spectra taken from different points of the matrix are presented to show the range of chemical composition and gel type. The gel marked as “2” with relatively high Si and Na with sufficient Ca and Al to form the C-N-A-S-H-type phase covered the lower area of the matrix. The appearance of light gray gel outlined above the dark gray gel (marked as “3”) is rich in Al and Ca, with negligible Si, indicating a C-A-H-type gel. This gel type could be structurally changed from CH as the Ca reacted with Al, thereby improving the cementitious properties and thus the strength. The third gel marked as “4” consists of relatively high Ca, Al and Si peaks with low Na, suggesting an alumina-rich C-A-S-H-type gel. As observed in Figure 11c, the sample WGA-20 F50S50 is composed of compact gel. Within the area shown in the micrograph with the 20 µm scale bar, no different gel phases were observed. The magnified micrograph with the 5 µm scale bar was taken from another area of the sample and shows two different gel types formed in the matrix. The gel marked as “1” covered most of the area of the sample, and is composed of rich Si and Ca and low Na and Al, resulting in increased Si/Al, suggesting low or/no Al-rich phases, thus making the structure chemically homogeneous and defining the Ca-rich C-A-S-H or C-S-H gel responsible for improved strength [47,50]. While, the gel marked as “2” in a matrix is composed of high Ca and Si but the Al and Na peaks are higher, making the gel chemically heterogeneous with increasing Al [50]. The microstructure of sample WGA-30 F50S50 in Figure 12d when compared with sample WGA-20 F50S50 in Figure 12c appears coarser, indicating the formation of a loose matrix due to excessive water in the system, resulting in lower compressive strength. This matrix is composed of three different gels, the porous gel “1”, suggesting Al-rich C-A-S-H or C-N-A-S-H gel, as it is found rich in Si and Al with sufficient Ca and Na covering most of the area of the matrix. The gel marked as “2” is rich in Si and Ca, indicating the formation of Si-rich C-S-H gel, which is believed to improve the strength, but its contribution to the strength development depends its formation per unit volume of matrix. It is not possible to measure the quantity of this gel in the matrix, but it is observed to occur less throughout the matrix. While, the maximum occurrence of the third gel marked as “3” was observed throughout the matrix, showing high Si and medium Ca, Al and Na, suggesting the formation of C-N-A-S-H gel. In conclusion, sample F50S50 showed the high leaching of elements from the precursors with increasing WGP content, i.e., from 0 to 30 g in NaOH solution, making the matrix denser and homogeneous.

With increasing GGBS content, i.e., 100% GGBS in FA/GGBS composites, the micrographs of sample F0S100 in Figure 13 appear to be denser with few or no undissolved GGBS particles compared to the F100S0 and F50S50 samples in Figure 11 and Figure 12, respectively. The matrix of sample WGA-0 F0S100 in Figure 13a exhibits loose gel with lower compressive strength than the F0S100 activated by WGA-10, WGA-20 and WGA-30. From the EDX analyses, it is observed that the Ca-based gel was present in two different compounds. The gel marked “1” composed of high Ca and medium Si indicates C-S-H, but with a high Ca/Si ratio. The scale-type gel marked as “2” is assumed to be a semi-crystalline phase, or an amorphous phase co-existing with the crystalline compound of Ca(OH)_2_ [51,52]. Compared to the sample WGA-0 F0S100 in Figure 13a, the sample WGA-10 F0S100 in Figure 13b exhibits a more compact matrix. The spectrum of gel marked as “1” suggests the formation of C-A-S-H- or C-N-A-S-H-type gels. The gel marked as “2” in sample WGA-10 F0S100 in Figure 13b is composed mainly of Ca and Si with low Al and Na, suggesting the formation of a C-S-H-type gel, which is the same as that observed in sample WGA-0 F0S100 but with increased Si, resulting in higher compressive strength than WGA-0 F0S100 [53]. The sample WGA-20 F0S100 in Figure 13c also exhibits a compact matrix, however the C-S-H gel marked as “2” shows increased concentration of Si and Ca compared to WGA-0- and WGA-10-activated F0S100. This indicates that, with increasing WGP content in NaOH solution, the leaching of Si and Ca from GGBS increased, forming the cross-linked structure responsible for improved strength (Figure 4 and Figure 5). The gel marked as “3” observed in the micrograph could be a modified version of the semi-crystalline gel observed in sample WGA-0 F0S100, as the concentration of glass increased in the activating solution, the crystallinity of hydrate phase reduced, further improving the compressive strength. As observed in Figure 13d, the sample WGA-30 F0S100 exhibits slightly loose gel throughout the matrix compared to samples WGA-10 F0S100 and WGA-20 F0S100. When the sample was activated by WGA-30 solution, additional water was supplied to the matrix, forming porous gel marked as “1”, which is composed of high Al and Si, as shown in the spectrum, and is responsible for reduced strength. It is worth noting that, when WGA-30 solution was used to activate the precursors, high levels of leaching of Al were observed to occur, which was available to form the gel. This phenomenon is assumed to be due to the hydrophilic nature of Al. However, when the precursor was 100% FA and activated by WGA-30, the gel observed consists of higher level of Al than Si (see Figure 11d). As the GGBS was incorporated in composites, i.e., WGA-30 F50S50 (see Figure 12d) and WGA-30 F0S100 (see Figure 13d), the peak of Si was observed to be higher than Al of a gel, indicating high reactivity of GGBS.

## 4. Conclusions

This study explored the effects of using NaOH with dissolved WGP to activate Class C FA/GGBS mixtures. It was revealed that, as the quantity of WGP in the NaOH solution increased, the Si concentration increased, which significantly enhanced the reactivity of the FA and GGBS precursor materials. As the Si level in the NaOH solution increased, the viscosity of the solution also increased, thereby leading to an increased water demand when mixing the FA/GGBS composites to obtain consistent workability. When the FA/GGBS composites were synthesized using WGA-10 and WGA-20 solutions, the presence of soluble silica in the solution, along with adequate water supplied to the system, accelerated the geopolymerization, resulting in increased strength and decreased porosity. Further increase in Si in the NaOH solution, i.e., WGA-30, corresponded to an even higher water demand, which led to the development of a more porous microstructure (as verified using porosity measurements and SEM micrographs), because the excess water accumulated in micropores after hardening, thereby reducing the strength. Furthermore, upon activation of FA/GGBS by the WGA-10, WGA-20 and WGA-30 solutions, the formation of amorphous phases increased (as verified through XRD), which was believed to be responsible for the strength development of the composites.

Owing to its amorphous nature, the GGBS was found to be superior to FA for improving the strength and reducing the porosity of the matrix. The presence of reactive Ca supplied from GGBS along with the alkaline solution containing dissolved silica, a homogeneous and compact matrix was developed with low porosity and high mechanical strength.

The nature of the alkaline solution, together with the ratio of FA/GGBS in the composites, played a significant role in forming the composition of the reaction product, as verified through SEM/EDX. In the FA-dominated composites, the main reaction product was N-A-S-H, which was initially formed in the FA-GGBS composites activated by the NaOH solution, i.e., WGA-0, and which was structurally and chemically modified when activated by WGA-10, WGA-20 and WGA-30 solutions, as dissolution of the precursor materials increased. Meanwhile, Ca-based hydration products, i.e., C-S-H or C-A-S-H, were observed when the GGBS level was increased in the composites, which were also structurally and chemically modified upon activation by the alkaline solution, namely WGA-10, WGA-20 and WGA-30.

## Figures and Tables

**Table 1:** Chemical composition of precursor materials and glass of three different colors.

**Precursor Material**								
**Elements (wt.%)**	**Si**	**Al**	**Na**	**Ca**	**Mg**	**Fe**	**O**	**K**
FA	18.16	10.33	0.65	18.72	0.26	2.01	43.44	1.53
GGBS	14.57	7.37	0.47	39.92	0.41	0.51	35.43	0.35
**Oxides (wt.%)**	**SiO_2_**	**Al_2_O_3_**	**Na_2_O**	**CaO**	**Fe_2_O_3_**		**K_2_O**	**MgO**
FA	38.84	19.52	0.87	26.19	2.87		1.84	0.43
GGBS	31.17	13.92	0.63	55.85	0.729		0.421	0.67
**Soda-Lime Waste Glass**								
**Elements (wt.%)**	**Si**	**Al**	**Na**	**Ca**	**Mg**	**Fe**	**O**	**K**
BG	34.05	2.61	6.92	8.46	1.49	0.47	35.62	0.72
GG	27.07	0.08	8.30	0.28	0.03	0.35	53.31	0.08
TG	39.57	2.99	9.39	5.87	0.69	0.21	37.27	0.54
**Oxides (wt.%)**	**SiO_2_**	**Al_2_O_3_**	**Na_2_O**	**CaO**	**Fe_2_O_3_**		**K_2_O**	**MgO**
BG	72.84	4.93	9.32	11.84	0.67		0.86	2.47
GG	57.90	0.15	11.18	0.39	0.60		0.09	0.05
TG	84.64	5.64	12.65	8.21	0.30		0.65	1.14

**Table 2:** Samples’ ID and mix proportion.

**Batch 1: WGA-0**	**Alkaline Solution: NaOH-4M**						
pH of WGA-0	12.5						
Specimens ID	F100S0	F85S15	F70S30	F50S50	F30S70	F15S85	F0S100
FA (wt.%)	100	85	70	50	30	15	0
GGBS (wt.%)	0	15	30	50	70	85	100
Liquid/solid	0.4						
**Batch 2: WGA-10**	**Alkaline Solution: NaOH-4M + 10 g (10 g/100 mL)**						
pH of WGA-10	12.55						
Specimens ID	F100S0	F85S15	F70S30	F50S50	F30S70	F15S85	F0S100
FA (wt.%)	100	85	70	50	30	15	0
GGBS (wt.%)	0	15	30	50	70	85	100
Liquid/solid	0.4						
**Batch 3: WGA-20**	**Alkaline Solution: NaOH-4M + 20 g (20 g/100 mL)**						
pH of WGA-20	12.51						
Specimens ID	F100S0	F85S15	F70S30	F50S50	F30S70	F15S85	F0S100
FA (wt.%)	100	85	70	50	30	15	0
GGBS (wt.%)	0	15	30	50	70	85	100
Liquid/solid	0.4						
**Batch 4: WGA-30**	**Alkaline Solution: NaOH-4M + 30 g (30 g/100 mL)**						
pH of WGA-30	12.47						
Specimens ID	F100S0	F85S15	F70S30	F50S50	F30S70	F15S85	F0S100
FA (wt.%)	100	85	70	50	30	15	0
GGBS (wt.%)	0	15	30	50	70	85	100
Liquid/solid	0.4						

**Table 3:** Elemental composition of waste glass-based alkaline solution (WGA).

Elements (ppm)	Si	Na	Al	Ca	Mg
WGA-10	3508	84,400	56.9	0.20	<0.1
WGA-20	6244	94,880	94.1	0.18	0.22
WGA-30	8752	97,840	106.3	0.41	5.82
